# Difference analysis of intestinal microbiota and metabolites in piglets of different breeds exposed to porcine epidemic diarrhea virus infection

**DOI:** 10.3389/fmicb.2022.990642

**Published:** 2022-11-01

**Authors:** Zhili Li, Wandi Zhang, Langju Su, Zongyang Huang, Weichao Zhang, Liangliang Ma, Jingshuai Sun, Jinyue Guo, Feng Wen, Kun Mei, Saeed El-Ashram, Shujian Huang, Yunxiang Zhao

**Affiliations:** ^1^College of Life Science and Engineering, Foshan University, Foshan, China; ^2^Guangxi Yangxiang Co., Ltd., Guigang, China; ^3^Liaoning Agricultural Development Service Center, Shenyang, China; ^4^Faculty of Science, Kafrelsheikh University, Kafr El-Sheikh, Egypt

**Keywords:** microbiota, metabolites, porcine epidemic diarrhea, Luchuan pig, Largewhite pig

## Abstract

The gut microbial composition of the Luchuan (LC) piglet, one of China’s native breeds, has rarely been studied, especially when compared to other breeds. This study developed a porcine epidemic diarrhea virus (PEDV) infection model in LC and Largewhite (LW) piglets, and analyzed the patterns and differences of intestinal microbial communities and metabolites in piglets of these two breeds after infection. The diarrhea score, survival time, and distribution of viral antigens in the intestine of piglets infected with PEDV differed among breeds, with the jejunal immunohistochemistry score of LW piglets being significantly higher than that of LC piglets (*P* < 0.001). The results of 16S rRNA sequencing showed differences in microbial diversity and community composition in the intestine of piglets with different breeds between PEDV infection piglets and the healthy controls. There were differences in the species and number of dominant phyla and dominant genera in the same intestinal segment. The relative abundance of *Shigella* in the jejunum of LC piglets after PEDV infection was significantly lower than that of LW piglets (*P* < 0.05). The key microorganisms differed in the microbiota were *Streptococcus alactolyticus*, *Roseburia faecis*, *Lactobacillus iners*, *Streptococcus equi*, and *Lactobacillus mucosae* (*P* < 0.05). The non-targeted metabolite analysis revealed that intestinal metabolites showed great differences among the different breeds related to infection. Spearman correlation analysis was conducted to examine any links between the microbiota and metabolites. The metabolites in the intestine of different breeds related to infection were mainly involved in arginine biosynthesis, synaptic vesicle cycle, nicotinic acid and nicotinamide metabolism and mTOR signaling pathway, with significantly positive or negative correlations (*P* < 0.05) between the various microorganisms. This study provides a theoretical foundation for investigating the application of core microorganisms in the gut of piglets of different breeds in the digestive tracts of those infected with PEDV, and helps to tackle the antimicrobial resistance problem further.

## Introduction

Diarrhea has long been a worldwide issue in piglet farming. Piglet diarrhea is caused by various factors, including nutrition and infectious agents. Porcine epidemic diarrhea virus (PEDV) is a leading cause of intestinal damage in piglets, causing watery diarrhea, vomiting, dehydration, and even death (Li et al., 2012; Sun et al., 2012; Song et al., 2015). The relationships between intestinal microbial dysbiosis and diseases have recently attracted the public’s interest. A growing body of research suggests that bacteria significantly influence gut barrier integrity (Mazmanian et al., 2005; Kamada et al., 2012; Manichanh et al., 2012). The intestinal microbiota of piglets is a complex system that constantly changes. Recent studies have linked intestinal barrier damage in diarrhea piglets to gut microbiota disorders (Kongsted et al., 2013; Hermann-Bank et al., 2015).

There were significant differences in the number and species of intestinal microbiota in diarrhea piglets compared to healthy piglets. In PEDV-infected piglets, the abundance of *Fusobacterium* and *Veillonella* increased in the intestinal microbiota (Koh et al., 2015; Liu et al., 2015; Huang et al., 2019). A recent study about Landrace-Yorkshire piglets infected with PEDV reported that the abundance of *Escherichia-shigella* was higher in infected piglets than in uninfected piglets, whereas the abundance of *Lactobacillus* was lower in the infected piglets (Dong et al., 2021). Another earlier study on gut microbiota in Duroc × Landrace × Large White piglets infected with PEDV showed that infection decreased the abundance of Shigella and increased the abundance of *Lactobacillus* (Wu et al., 2020). Scholars have provided a great deal of research regarding the relationship between intestinal microbiota and different diseases in recent years (Lindberg, 2014; H. Yang et al., 2018). Diet and environment also have a noticeable effect on the composition of swine intestinal microbiota (Haenen et al., 2013; Gresse et al., 2017). Additionally, differences in the types of intestinal microorganisms may exist between swine breeds or geographical locations (L. Yang et al., 2014; Cheng et al., 2018; Xiao et al., 2018).

Many studies have shown that certain microbiota members in mammal digestive tracts can help treat gastrointestinal problems. Others have used these members to treat inflammatory bowel disease, irritable bowel syndrome, and other diseases that can reduce the need for antibiotics and other medications (Amit-Romach et al., 2008; Gkouskou et al., 2014; Tomasello, 2014; Imperatore et al., 2017). Early weaning of piglets could significantly shorten the animal husbandry cycle in current livestock. Nonetheless, its stress frequently results in diarrhea, and newly introduced commercial piglets are more susceptible to intestinal stress than indigenous breeds. Increases in morbidity, mortality and economic cost can result from early weaning (Gresse et al., 2017; Wang et al., 2019).

The Luchuan (LC) pig is a Chinese domestic breed with small body size, good maternal and reproductive performance, and high-quality meat (Ran et al., 2014). Since antibiotics have been explicitly banned in the EU livestock industry, finding alternatives to antibiotic treatment was essential to address intestinal stress in piglets. Therefore, this study investigated the changes of intestinal microorganisms and metabolites in LC and Largewhite (LW) piglets related to PEDV artificial infection. Meanwhile, microorganisms and metabolites related to infection in different breeds of piglets were screened, and correlations were searched to help find suitable probiotics that would provide treatments for piglet diarrhea caused by PEDV.

## Materials and methods

### Experimental design

Two sows (*Sus scrofa domestica*) with the same farrowing date were chosen at a small pig farm in Guangxi province. One was a LW sow, while the other was a LC sow, a local breed unique to Guangxi province. On farrowing day, 12 piglets with an initial average body weight of 1.28 ± 0.03 kg were chosen at random, six for each group, and gender was balanced in the various groups. The umbilical cord of each piglet was trimmed and the navel disinfected with iodine. The piglets did not consume any colostrum or antibiotics and after determining good health were transferred to an experimental animal house where they were allocated into groups according to breed differences. The 12 piglets were divided into four groups (*n* = 3): LW piglets challenged with PEDV (PEDV LW group), LC piglets challenged with PEDV (PEDV LC group), LW piglets control (Control LW group), and LC piglets control (Control LC group). Three-day old piglets were gavaged with 2 ml of PEDV at a titer of 10^3^ TCID_50_. PEDV strain JS-13 used in this work was provided by Shanghai Veterinary Research Institute, Chinese Academy of Agricultural Sciences. The virulence of the PEDV strain was evaluated in the preliminary intracellular growth assay. The dose of challenge estimates for animal experiments were based on prior animal vivo assays within the laboratory for determination of the optimum infective dose. The control LW and control LC groups were left unchallenged. Clinical signs such as diarrhea score and fecal shedding were monitored daily following the challenge. Before and after the challenge, feces swabs were collected from each pig at 12 h before the challenge and 12, 24, 44, 63, 84, and 96 h after the challenge until the end of the study. All feces swabs were stored in centrifuge tubes and frozen immediately on ice until transferred to −80°C storage. After the last piglet in the PEDV group died, six piglets in the control group were euthanized.

### Determination of viral loads by real-time quantitative polymerase chain reaction

Real-time polymerase chain reaction (PCR) primers and probes were designed using Primer 5 software to amplify the M gene from PEDV (Table 1). Primers were used to amplify the M fragment using synthesized cDNA extracted from PEDV positive materials as a template. The amplified products were separated using agarose gel electrophoresis, and the target fragments were cut out and purified under UV light. The purified product was ligated with pGEM-T for 2 h at 16°C before being transformed into *E. coli* DH5α competent cells. A single colony was chosen from the plate and placed in LB broth medium. After overnight incubation (37°C for 4 h), plasmids were extracted from positive clones using Thermo Scientific GeneJET Plasmid Miniprep Kit, identified by PCR, and sequenced.

**TABLE 1:** Primers for porcine epidemic diarrhea virus (PEDV) detection.

Primer	Sequence (5′-3′)	Primer length (bp)
PEDV-F	CGTACAGGTAAGTCAATTAC	20
PEDV-R	GATGAAGCATTGACTGAA	18
PEDV-Pro	FAM-TTCGTCACAGTCGCCAAGG-BHQ1	19

A spectrophotometer was used to determine the concentration and purity of the extracted plasmid and the gene copy number. A standard plasmid was used as a template, a 10-fold gradient dilution was performed, and three repeats of each plasmid concentration were made. Simultaneously, fecal samples and a negative control were run, and the reaction was carried out on a Roche fluorescence quantitative PCR. The reaction system was 20 μL, including ddH_2_O 7.6 μL, 2 × Ace qPCR Probe Master Mix10 μL, primer PEDV-F 0.4 μL, primer PEDV-R 0.4 μL, probe 0.2 μL, 50 × ROX Reference Dye1 0.4 μL, template 1 μL. The reaction conditions were as follows: 95°C, 5 min, 95°C, 10 s, 60°C, 30 s, 40 cycles. The fluorescence signal was collected during extension, and the kinetic and standard curves were obtained.

### Histological observation and immunohistochemistry

Following inoculation, piglets from the control group were sacrificed on day 7, at which point all piglets in the infected groups had died of diarrhea. Their intestines were quickly removed after they died. Intestinal sections from the middle jejunum and cecum were collected and immediately fixed in 10% buffered formalin for 24 h. The tissues were removed from the formalin solution and dehydrated in graded alcohol solutions before being embedded in paraffin. The embedded-paraffin tissue samples were then cut into 4 μm sections and stained with hematoxylin and eosin (H&E). Immunohistochemistry (IHC) staining was performed with monoclonal antibody against PEDV-N protein on these samples as described previously (Curry et al., 2017a). The villus height (VH) and crypt depth (CD) of jejunum were performed using Image J software at × 100 magnification.

### DNA extraction and 16S rRNA amplicon sequencing

Jejunal and cecal content samples used in microbiota analysis came from 12 piglets, as shown in Table 2. Total DNA was extracted from the jejunal and cecal contents in sterile centrifuge tubes using QIAmp DNA kit (Qiagen, Germany) according to the manufacturer’s instructions. The V3-V4 hypervariable regions of the bacterial 16S rRNA gene were amplified using the primers 338F-ACTCCTACGGGAGGCAGCAG and 806R-GGACTACHVGGGTWTCTAAT. According to the manufacturer’s protocol, the PCR products were confirmed on a 2% agarose gel, purified using an AxyPrep DNA Gel Extraction Kit AP-GX-250 (Axygen Biosciences, Union City, CA, United States), and quantified using QuantiFluorTM-ST (Promega, Madison, WI, United States). PCR-purified amplicons were pooled in equimolar amounts and paired-end sequenced (2 × 300 bp) on the Illumina NovaSeq platform, according to standard protocols from Shanghai Personal Biotechnology Co., Ltd., (Shanghai, China) (Illumina, San Diego, CA, United States). This study’s 16S rRNA gene sequence data were deposited in the GenBank Sequence Read Archive database under SRA: SRP346825.

**TABLE 2:** The information of samples collected for microbiota analysis.

The group name of the samples	Sample type	Number
Con LC J	Jejunal content	3
Con LW J	Jejunal content	3
PEDV LC J	Jejunal content	3
PEDV LW J	Jejunal content	3
Con LC C	Cecal content	3
Con LW C	Cecal content	3
PEDV LC C	Cecal content	3
PEDV LW C	Cecal content	3

### Untargeted metabolomic analysis

After dissection, the jejunal and cecal tissues (Table 3) were quickly frozen in liquid nitrogen. The tissue was then cut on dry ice (10 mg) and placed in a microcentrifuge tube (2 mL). The tissue samples with 200 μL of sterile H_2_O were homogenized using the Benchmark Beadbug homogenizer (Benchmark Scientific, United States). 800 μL of methanol/acetonitrile (1:1, v/v) was added to the homogenized solution for metabolite extraction. The mixture was centrifuged for 15 min (4°C, 14,000 × *g*). The supernatant was dried in a vacuum centrifuge. The samples were re-dissolved in 100 μL of acetonitrile/water (1:1, v/v) solution for LC-MS analysis. Analyses were performed using a UHPLC (1290 Infinity LC, Agilent Technologies) coupled to a quadrupole time-of-flight (AB Sciex TripleTOF 6600) at Shanghai Applied Protein Technology Co., Ltd.

**TABLE 3:** The information of samples collected for microbiota analysis.

The group name of the samples	Sample type	Number
Con LC J	Jejunal tissue	3
Con LW J	Jejunal tissue	3
PEDV LC J	Jejunal tissue	3
PEDV LW J	Jejunal tissue	3
Con LC C	Cecal tissue	3
Con LW C	Cecal tissue	3
PEDV LC C	Cecal tissue	3
PEDV LW C	Cecal tissue	3

### Statistical analysis

*T*-tests and one-way analyses of SPSS software 19.0 were used to compare between-group differences and groups (SPSS, Inc.). ^**^P < 0.01 or ΔΔ*P* < 0.01 was considered extremely significant, while *P < 0.05 or Δ*P* < 0.05 was considered significant.

## Results

### Differences in clinical indicators after porcine epidemic diarrhea virus infection in piglets

**FIGURE 1:**
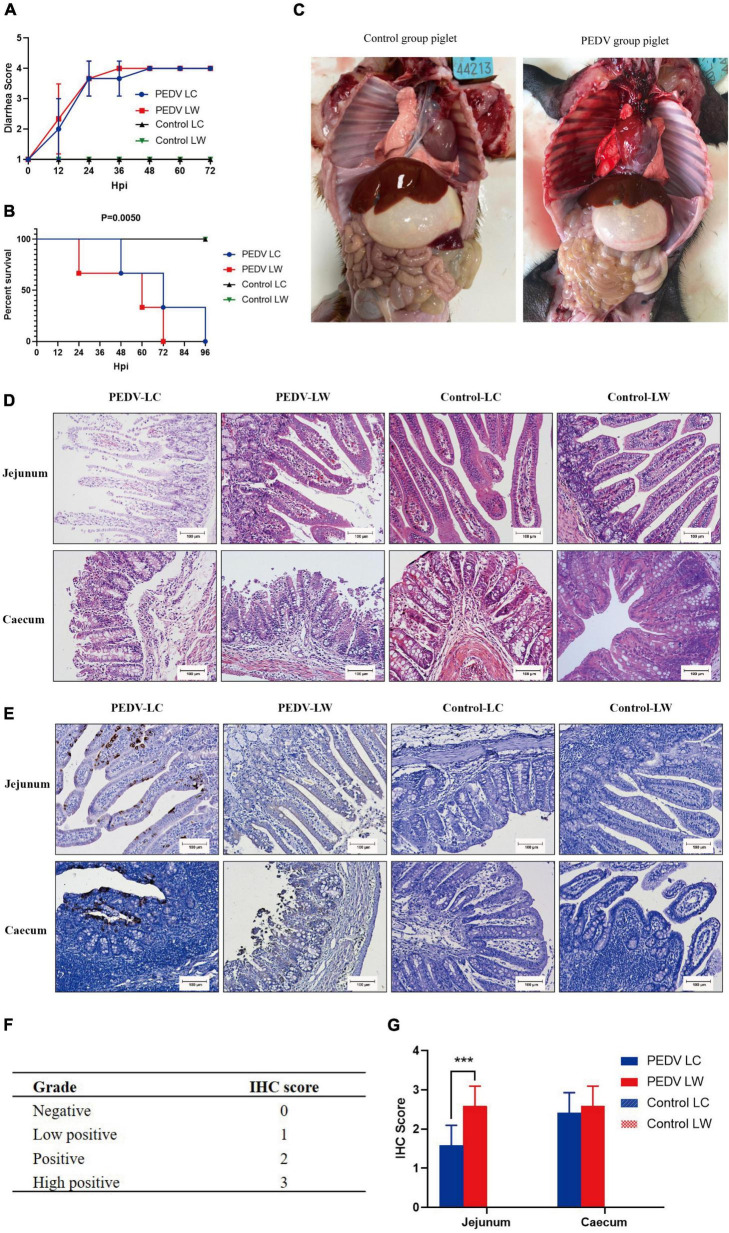
**(A)** Diarrhea score of piglets. **(B)** Survival time of piglets. **(C)** Piglet autopsy lesions. **(D)** Intestinal H&E staining (100 μm). **(E)** Immunohistochemical staining (100 μm). **(F)** Immunohistochemistry scoring criteria. **(G)** Immunohistochemistry score.

Within 12 h of receiving PEDV orally, some piglets in the infected group had feces with a watery appearance and a fishy odor. Some even displayed symptoms, such as vomiting and difficulty breathing. The first piglet that showed symptoms, which belonged to the PEDV LW group, died within 24 h of infection. Then, within 24∼96 h, both infected piglets in the PEDV LC and PEDV LW groups died. Each piglet was scored and averaged daily by observing fecal morphology, color, and bleeding (Table 4). The results showed that the piglets in the PEDV LC group had lower diarrhea scores and survived longer than the piglets in the PEDV LW group (Figures 1A,B). The plasmid standards amplification plot of Ct vs. log concentration was used to generate a standard curve (slope = −3.2782). After fitting the data to the standard curve, the fecal viral load values measured in the infected groups were shown in Table 5. The fecal gene copy number content was lower in the PEDV LC group than in the PEDV LW group. However, the difference was insignificant.

**TABLE 4:** Fecal grading.

Morphology of feces	Score
Normal and shaped feces	1
Pasty feces and not shaped	2
Semi-liquid diarrhea with some solid content	3
Liquid diarrhea with no solid content	4

**TABLE 5:** Mortality and viral load of porcine epidemic diarrhea virus in feces.

Hours post infection (Hpi)	Number mortality		Mean log (genomics copies/μL)	
		
	PEDV LC	PEDV LW	PEDV LC	PEDV LW
12	0	0	4.585	5.278
24	0	1	5.000	5.045
44	0	1	5.020	5.127
63	2	2	5.015	4.841
84	2	3	5.027	–
96	2	3	4.809	–

### Histological and pathological differences in piglets infected with porcine epidemic diarrhea virus

Piglets from the two infected groups had different degrees of partial swelling of the small intestine, transparency of tissue of the intestinal wall, narrowing of the intestinal lumen, and individual blood spots after their abdominal cavities were dissected for visual inspection (Figure 1C). Tissue sections stained with H&E revealed that all piglets in the infected group had intestinal histopathological changes. The small intestinal villi in the jejunal section were damaged and lysed, and the cytoplasm appeared vacuolated. The jejunum epithelial cells were necrotic and detached, showing evidence of cell necrosis in the lamina propria. Furthermore, the cecal lesions were characterized primarily by dilated blood vessels, the indistinguishable structure of the large intestinal glands, and the disintegration and shedding of many intestinal gland cells. There were also a few lymphocytes in the cecum submucosal layer (Figure 1D). Piglets from infected groups had decreased VH to CD ratio in the jejunum (*P* < 0.05), lower VH, and tented to have decreased CD compared with the control group (Table 6).

**TABLE 6:** Effects of porcine epidemic diarrhea virus on jejunal morphology of piglets.

Items	Con LW	Con LC	PEDV LW	PEDV LC	*P*-value
Villus height, μm	1229.299	926.0753	455.7345	479.8395	0.0265
Crypt depth, μm	319.7322	208.9695	154.1545	117.6867	0.02
Villus height/Crypt depth	3.844778	4.590073	3.093606	4.354744	0.0012

The distribution of PEDV in the intestine was characterized by immunohistochemical staining, which showed the presence of viral antigens in both the jejunum and cecum of the experimental group (Figure 1E). Viral antigens were mainly present in the cytoplasm and lamina propria of the jejunal villous epithelium. A small number of antigens were also present in the intestinal glands of the cecum. Immunohistochemical scoring of intestinal tissue sections using Image J software according to the scoring criteria in Figure 1F showed differences in the distribution of PEDV N protein antigens in the jejunum and cecum between the PEDV LC and PEDV LW groups (Figure 1G). Also, there were significantly higher immunohistochemical scores in the jejunum of the PEDV LW group than in the PEDV LC group (*P* < 0.001). Furthermore, the PEDV LW group had more severe intestinal histopathology than the PEDV LC group and contained more antigens, as evidenced by H&E and IHC scores.

### Characterization of the intestinal microbiota composition of piglets after exposure to porcine epidemic diarrhea virus

After filtering and denoising, 1,712,473 sequence reads remained out of 1,934,068 raw sequences obtained from 24 samples (including 12 jejunal and 12 cecal content samples). On average, the sequence reads were 427 bp (16–441 bp). The average number of OTUs identified after species-specific taxonomic annotations were 499 OTUs, including 232 phyla, 81 classes, 154 orders, 850 families, 2,145 genera, 3,125 species and one unclassified species.

**FIGURE 2:**
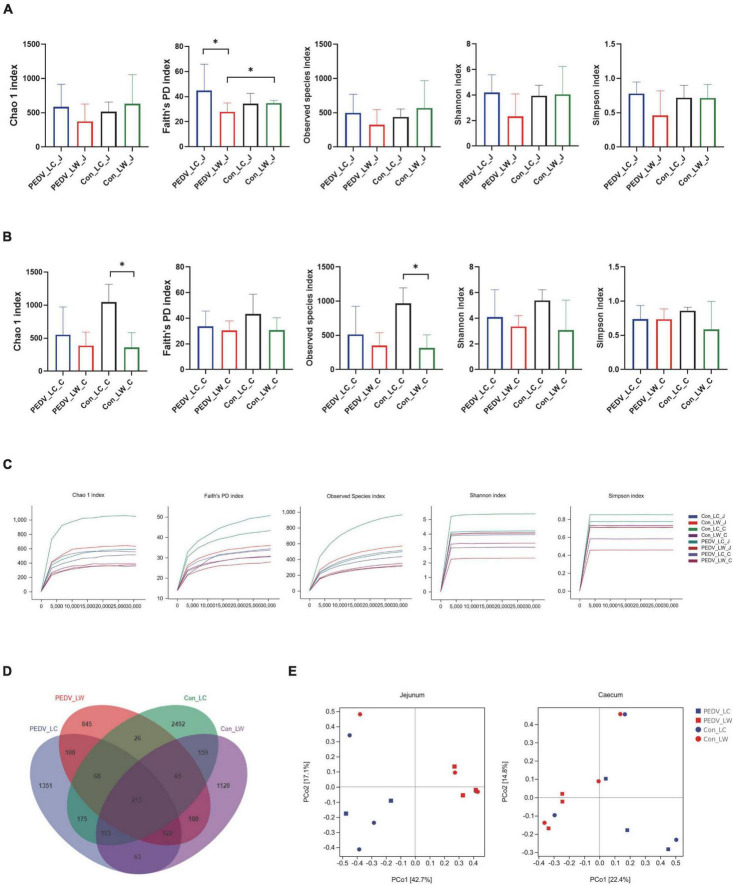
**(A)** Microbial alpha diversity in the jejunum. **(B)** Microbial alpha diversity in the cecum. **(C)** Rarefaction curves of alpha diversity. **(D)** OTU venn diagram. **(E)** Principal coordinate analysis (PCoA) of intestinal microorganisms in piglets exposed to porcine epidemic diarrhea virus (PEDV) infection.

The alpha diversity results revealed a significant difference between the PEDV LC and PEDV LW groups using the Chao1, Faith’s PD, Observed species, Shannon and Simpson index in the jejunum and cecum (Figures 2A,B). Compared to the control LC group, the Chao1 and Observed species index in the cecum showed a statistically significant decrease in the control LW group (Figure 2B). The results of the flattened sparse curve show that the number of samples required for 16S rRNA analysis was achieved (Figure 2C).

According to the Venn diagram, PEDV infection decreased the number of OTUs in the jejunum and cecum of piglets from 4,724 to 3,241. Interestingly, the number of species-specific OTUs in the intestines of LC piglets was always higher than that of LW piglets whether infected with PEDV or not (Figure 2D). Furthermore, the differences in microbial community composition between the two breeds were investigated using principal coordinate analysis (PCoA) based on the Bray-Curtis metric. Overall, the LC and LW piglet groups were split into two distinct communities clustered together (Figure 2E).

### Screening for species with intestinal differences in piglets of different breeds exposed to porcine epidemic diarrhea virus

**FIGURE 3:**
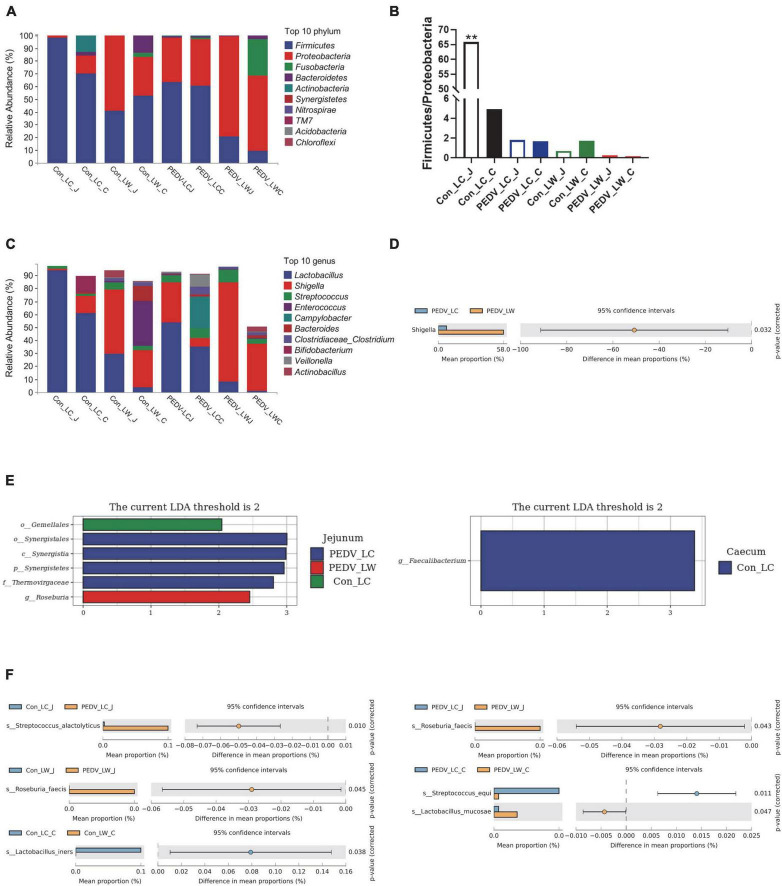
**(A)** Phylum-level species composition. **(B)** Ratio of firmicutes and proteobacteria. **(C)** Genus-level species composition. **(D)** Differences between groups at the genus level. **(E)** LDA values of linear discriminant effect size (LEfSe) analysis. **(F)** Intergroup differences at the species level.

Differences in species composition between groups at the phylum and genus levels were described. Firmicutes and Proteobacteria were the most abundant phyla in the jejunal and cecal communities, followed by Bacteroidetes, Actinobacteria, and Fusobacteria (Figure 3A). PEDV infection caused a decrease in the ratio of Firmicutes to Proteobacteria in the jejunum and cecum, with the percentage of Firmicutes to Proteobacteria in the jejunum of the control LC group being significantly higher than in the other groups (Figure 3B). At the genus level, the abundance of *Lactobacillus* in the jejunum and cecum showed a decreasing trend after infection with PEDV, with the abundance of *Shigella* and *Streptococcus* showed an increasing trend. Notably, the relative abundance of Lactobacillus was consistently higher in LC piglets than in LW piglets whether infected with PEDV or not. In comparison, the relative abundance of *Shigella* was consistently lower in LC piglets than in LW piglets (Figure 3C). Furthermore, Welch’s *t*-test results showed that the relative abundance of *Shigella* was significantly higher in the PEDV LW group than in the PEDV LC group (Figure 3D).

Linear discriminant Effect Size (LEfSe) analysis with LDA scores > 2.0 was used to screen the bacteria further to identify microbial differences above the genus level exposed to PEDV infection (Figure 3E). *Gemellales*, *Synergistales*, and *Roseburia* were significantly different between groups (LDA > 2, *P* < 0.05). *Faecalibacterium* was significantly enriched in the cecum of the Con_LC group (LDA > 2, *P* < 0.05). Different microorganisms between subgroups were compared two-by-two and identified species levels using Metastats analysis (Figure 3F). A total of five significantly different species were obtained after performing the Welch’s *t*-test, *Streptococcus alactolyticus*, *Roseburia faecis*, *Lactobacillus iners*, *Streptococcus equi*, and *Lactobacillus mucosae* (*P* < 0.05). *Lactobacillus iners* was the dominant bacterium significantly enriched in the cecum of pre-infected piglets (Con LC group). In contrast, the remaining four different species were significantly increased in the jejunum and cecum of PEDV-infected piglets (*P* < 0.05).

### Metabolite changes caused by exposure to porcine epidemic diarrhea virus

**FIGURE 4:**
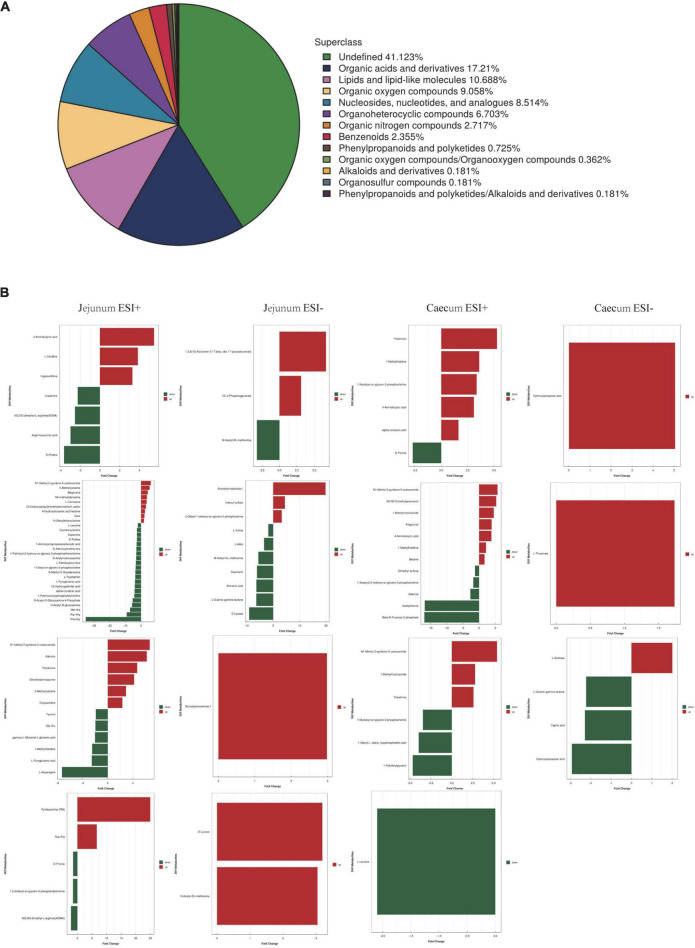
**(A)** Chemical classification of metabolite composition. **(B)** Fold change analysis of different metabolites.

To better understand the metabolite changes in the intestine of piglets of different breeds exposed to PEDV infection, all metabolites in the samples were identified by the UHPLC-MS platform. The results showed that 552 metabolites were identified, with 13 main categories according to their chemical classification. The top three categories were organic acids and derivatives (17.21%), lipids and lipid-like molecules (10.688%) and organic oxygen compounds (9.058%) (Figure 4A).

Supervised multivariate analyses were performed to show differences between groups by using principal component analysis (PCA) and orthogonal partial least squares discriminant analysis (OPLS-DA). PCA plots showed significant separation between sample groups and overlap of QC samples (Supplementary Figure 1A). OPLS-DA score plots showed substantial differences in intestinal metabolic profiles of piglets exposed to PEDV infection, and significant separation of intestinal metabolites occurred between LC and LW piglets with or without PEDV infection (Supplementary Figure 1B). The evaluation parameters R^2^Y and Q^2^ of the permutation test showed good stability in all OPLS-DA models (Supplementary Figure 1C).

**FIGURE 5:**
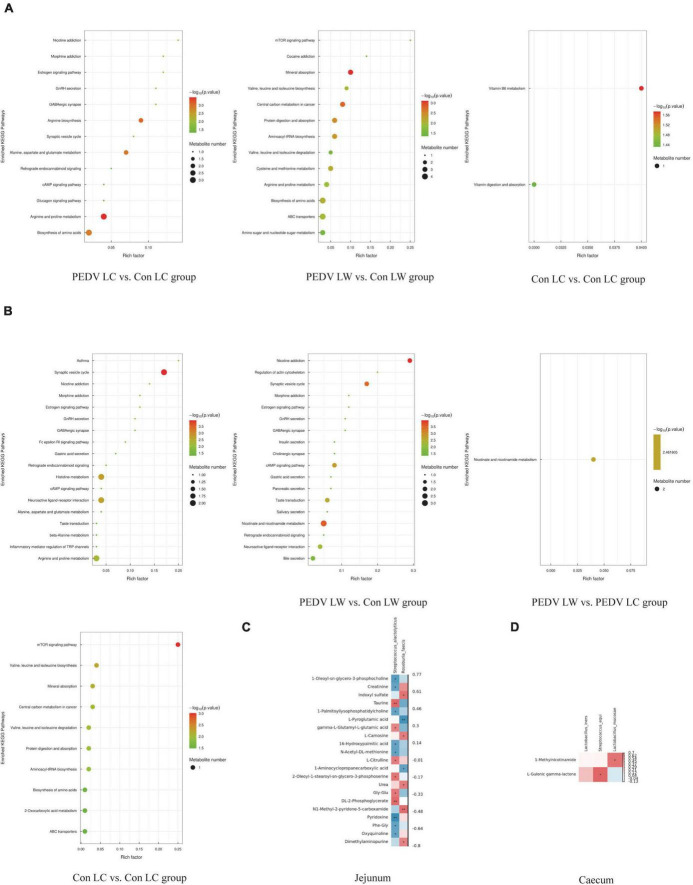
**(A)** KEGG enrichment pathway of different metabolites in jejunum. **(B)** KEGG enrichment pathway of different metabolites in cecum. **(C)** Spearman correlation analysis of different microorganisms and metabolites in the jejunum. **(D)** Spearman correlation analysis of different microorganisms and metabolites in the cecum.

The OPLS-DA model’s Variable Importance for Projection (VIP) values were used to identify the metabolites that contributed significantly to the model. As a result, 101 different metabolites were identified in jejunal and cecal samples using the OPLS-DA VIP > 1 and *P* < 0.05 criteria. The differential metabolite expression between groups was calculated using differential multiplicity analysis and visualized with histograms, and fold change (FC) values (FC > 1 for upward adjustment, FC < 1 for downward adjustment) that could be used as criteria for metabolite up-and down-regulation (Figure 4B). Of the 70 different metabolites identified in the jejunal samples, 28 were up-regulated, and 42 were down-regulated (Supplementary Table 1). 19 of the 31 different metabolites were significantly up-regulated, and 12 were significantly down-regulated in the cecum samples (Supplementary Table 2). All the different metabolites were matched against the KEGG database to obtain information on higher metabolite enrichment pathways (Figures 5A,B). In the jejunal content samples, certain metabolites were mainly associated with arginine and proline metabolism, arginine biosynthesis, mineral absorption, and vitamin B6 metabolism (Table 7, *P* < 0.05). Certain metabolites in cecum content samples were mainly enriched in the synaptic vesicle cycle, nicotinic acid and nicotinamide metabolism, and the mTOR signaling pathway (Table 8, *P* < 0.05).

**TABLE 7:** Significant enrichment pathways for jejunal differential metabolites.

Group	Pathway name	*P*-value
PEDV LC vs. Con LC group	Arginine and proline metabolism	0.000332
	Arginine biosynthesis	0.000802
PEDV LW vs. Con LW group	Mineral absorption	0.000796
Con LW vs. Con LC group	Vitamin B6 metabolism	0.026662

**TABLE 8:** Significant enrichment pathways for differential metabolites in the cecum.

Group	Pathway name	*P*-value
PEDV LC vs. Con LC group	Synaptic vesicle cycle	0.000114
PEDV LW vs. Con LW group	Nicotinate and nicotinamide metabolism	0.000246
PEDV LW vs. PEDV LC group	Nicotinate and nicotinamide metabolism	0.003455
Con LW vs. Con LC group	mTOR signaling pathway	0.000962

### Correlation between intestinal microbiota and metabolites that differ significantly

Spearman correlation analysis revealed a significant association between different intestinal microbiota and different metabolites in breeds of piglets infected with the PEDV or not (Figure 5). *S. alactolyticus* was negatively correlated with Pyridoxine, 1-Oleoyl-sn-glycero-3-phosphocholine, Creatinine, 1-Palmitoyllysophosphatidylcholine, 16-Hydroxypalmitic acid, N-Acetyl-DL-methionine, Phe-Gly and Oxyquinoline while positively correlated with DL-2-Phosphoglycerate, Taurine, gamma-L-Glutamyl-L-glutamic acid, L-Citrulline, 2-Oleoyl-1-stearoyl-sn-glycero-3-phosphoserine and Gly-Glu. *R. faecis* was negatively correlated with L-Pyroglutamic acid and 1-Aminocyclopropanecarboxylic acid while positively correlated with N1-Methyl-2-pyridone-5-carboxamide, Indoxyl sulfate, L-Carnosine and Urea. *S. equi* was positively correlated with L-Gulonic gamma-lactone, while *L. mucosae* was positively correlated with 1-Methylnicotinamide.

## Discussion

Characteristics of intestinal microbiota in pigs of various breeds were different in terms of features and composition. In a study completed recently, scientists found that the beta diversity of gut microbes was different between Jinhua (Chinese panda pigs) and commercial pigs (Duroc × Landrace × Yorkshire) (Curry et al., 2017b). Another recent study discovered similar differences in the intestinal microbiota composition between Duroc and Iberian pigs (López-García et al., 2021). The study of native Chinese pigs is ongoing. However, there are no reports on the characteristics of intestinal microbiota and metabolites in LC pigs. Some studies have reported that PEDV infection modified porcine gut microbial composition (Tan et al., 2019). Therefore, this study first compared the intestinal microbiota and gut metabolites between PEDV-infected and uninfected LC and LW pigs to understand their compositions and relationships further.

This study showed symptoms consistent with acute PEDV infection, such as lethargy and diarrhea, in as little as 6 h. After a while, the symptoms worsened with vomiting, dehydration and watery diarrhea. The first piglets died within 24 h, with the earliest fecal shedding occurring within 12 to 24 h after exposure to PEDV infection at the onset of symptoms. The histopathological findings revealed intestinal villi atrophy, severe damage to the villi, and epithelial cell exfoliation. Villous epithelial cells had brown PEDV antigen signals in their cytoplasm (Jung et al., 2015; Lin et al., 2015; Xu et al., 2020). The findings of these studies agreed with the observations of disease symptoms in this study. The IHC score and survival time of the LC piglet group were slightly different from those of the LW group in our results, but the differences were not significant due to the small sample size or the younger piglet age.

The present study evaluated the LC and LW piglets’ intestinal microbiota displayed different bacterial community characteristics, as evaluated by alpha diversity and PCoA analysis. We found higher species richness of cecum in the LC group than in the LW group (Chao1 index and Observed species). These data revealed microbial community diversity and complexity in different piglets gut systems. Besides, differences were found in the dominant phylum, and dominant bacterial genus in the same intestine of piglets in the LC and LW groups, and the cecum of piglets of the same breed was always richer in bacterial species than the jejunum, regardless of whether they were infected with PEDV or not. Firmicutes and Proteobacteria were the dominant phyla with relative abundance greater than 1% in the jejunum of piglets whether infected with the PEDV or not, Bacteroidetes, Actinobacteria and Fusobacteria increased in the number of dominant phyla in the cecum. Three dominant genera (*Lactobacillus*, *Shigella*, *Streptococcus* and *Actinobacillus*) with relative abundance greater than 5% were identified in the jejunum. The number of dominant genera with relative abundance greater than 5% in the cecum increased to eight (*Lactobacillus*, *Shigella*, *Streptococcus*, *Shigella*, *Streptococcus*, *Campylobacter*, *Bacteroides*, and *Bifidobacterium*). Previous studies had shown high microbial diversity in the large intestinal segment, with no microorganisms in abundance above 8% at the genus level and a more complex microbial composition in the distal intestinal part than in the proximal one, which was similar to the results of this study (Martinez-Guryn et al., 2019).

The abundance of species composition showed significant differences between the control and the PEDV infected piglets. Significant changes in the microbiota composition at the genus level were found in the jejunum and cecum. The Con LC and the PEDV LC groups displayed a higher abundance of Lactobacillus and a lower abundance of Shigella than the Con LW and the PEDV LW groups in the jejunum and cecum. Many studies suggest that *Lactobacillus* sp. helps to reduce pro-inflammatory cytokine production and enhance the antiviral immune response (Waki et al., 2014; Niederwerder, 2017). *Shigella* sp. has been shown to be a common cause of bacterial deaths, and most cases present with watery diarrhea and dysentery (MacLennan and Steele, 2022). *Fusobacterium* and *Shigella* abundances increased significantly after PEDV infection, while *Psychrobacter, Prevotella*, and *Faecalibacterium* abundances decreased significantly. Others have previously made a similar observation (Afra et al., 2013; Croxen et al., 2013). Because these symbiotic bacteria were known to play an important role in maintaining homeostasis, their reduction in the control group may negatively impact them (Koh et al., 2015). PEDV infection reduced probiotics and increased pathogenic bacteria, disrupting the intestinal microbiota structure (Song et al., 2017; Huang et al., 2019). These results suggest that PEDV infection caused profound alterations in piglets’ gut microbiota composition. However, the gut microbiome composition of LC pigs was significantly different from that of LW piglets, especially in the trend of changes in the microbiome composition of PEDV LC groups.

In this study, the critical microorganisms screened for differences in microbiota structure before and after infection in different breeds of piglets were *Streptococcus alactolyticus*, *Roseburia faecis*, *Lactobacillus iners*, *Streptococcus equi* and *Lactobacillus mucosae*. The significantly different microorganism *R. faecis*, belonging to the genus *Roseburia*, a butyric acid-producing bacterium, was identified in this experiment (Duncan et al., 2006), The increased content of acetic and volatile fatty acids in the cecum was beneficial for improving intestinal function and health (Duncan et al., 2006). *L. mucosae* was first isolated from the small intestine of pigs and was an anaerobic bacterium that ferments whey to metabolize L-lactate (Guerra et al., 2001). It is not clear whether the microorganisms screened for differences between groups are related to the process of PEDV infection in the host. To help clarify this, their mechanisms of action need to be further explored and elucidated. In this experiment, the metabolite 1-Methylnicotinamide that positively correlated with *L. mucosae* was upregulated in the cecum of the PEDV_LW group, indicating that the expression of this metabolite may be associated with the increased abundance of *L. mucosae*. *L. mucosae* belong to the family *Lactobacillaceae* and play an important role in gastrointestinal health (Walter, 2008; Delgado et al., 2015), was first identified by Roos et al. (2000), with probiotic properties, high viability, acid, and bile tolerance (de Palencia et al., 2011). *L. mucosae* LM1 had affinities for mucin glycan receptor analogs against pathogens such as *Escherichia coli* K88 and *Salmonella Typhimurium* KCCM 40253. This activity was demonstrated *in vitro* (Valeriano et al., 2014, 2016). 1-Methylnicotinamide was the active endogenous metabolite of nicotinamide (Alston and Abeles, 1988), involved in the nicotinic acid and nicotinamide metabolic pathways, possessed with anti-inflammatory characteristics (Gebicki et al., 2003; Bryniarski et al., 2008), and protective characteristics benefitting the gastrointestinal tract (Brzozowski et al., 2008). The specific relationship between 1-Methylnicotinamide and *L. mucosae* and whether both played a key role in the anti-PEDV infection process needs further experimental validation.

The differences in gut microbes and metabolites between the two piglet breeds observed in our study may be related to differences in the disease symptoms following PEDV infection. However, it is important to determine if there is a connection between the specific core microbiota found in LC piglets (microbiota that stays in the body with infection) and the PEDV infection process. One limitation of this study is the relatively small sample size. Future studies with a large sample size are desired to validate the discoveries in this study.

## Data availability statement

The datasets presented in this study can be found in online repositories. The names of the repository/repositories and accession number(s) can be found in the article/Supplementary material.

## Ethics statement

This animal study was reviewed and approved by Animal Protection and Ethics Committee and Use Committee of Foshan University.

## Author contributions

WeZ, YZ, WaZ, and ZL designed the study. ZH, LM, and LS performed the research and analyzed the data. WaZ wrote the initial draft of the manuscript. JG, FW, KM, SE-A, SH, and ZL revised the manuscript. All authors contributed to the article and approved the submitted version.
