# Supplementary material for: Difference analysis of intestinal microbiota and metabolites in piglets of different breeds exposed to porcine epidemic diarrhea virus infection
Source: Front Microbiol. 2022 Nov 1;13:990642. doi: 10.3389/fmicb.2022.990642 (PMC9665409; doi:10.3389/fmicb.2022.990642)
Supplement: Supplementary file 1 [file Data_Sheet_1.ZIP › supplementary meterials/supplementary figure1.docx]

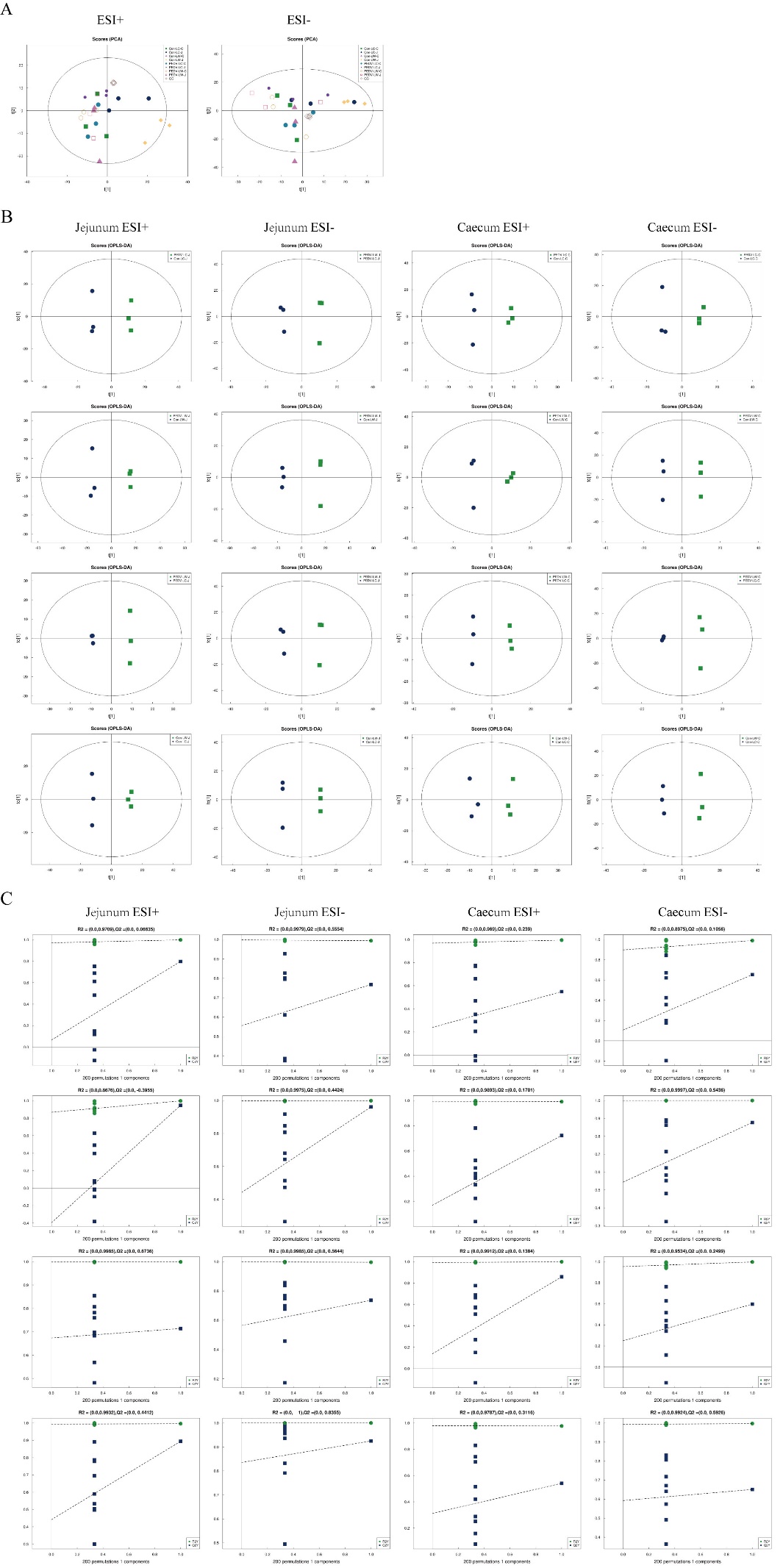


Figure S1 (A) PCA of jejunum and caecum samples in all groups. (B) OPLS-DA of jejunum and caecum samples in all groups. (C) Permutation tests in feces samples and liver tissues samples.

OPLS-DA (jejunum ESI+): R^2^X=0.494, R^2^Y=0.998, Q^2^=0.797 (PEDV-LC-J vs Con-LC-J); R^2^X=0.673, R^2^Y=0.997, Q^2^=0.946 (PEDV-LW-J vs Con-LW-J); R^2^X=0.74, R^2^Y=0.996, Q^2^=0.894 (Con-LW-J vs Con-LC-J); R^2^X=0.746, R^2^Y=0.999, Q^2^=0.714 (PEDV-LW-J vs PEDV-LC-J);OPLS-DA (jejunum ESI-): R^2^X=0.76, R^2^Y=0.994, Q^2^=0.768 (PEDV-LC-J vs Con-LC-J); R^2^X=0.782, R^2^Y=0.999, Q^2^=0.961 (PEDV-LW-J vs Con-LW-J); R^2^X=0.888, R^2^Y=1, Q^2^=0.925 (Con-LW-J vs Con-LC-J); R^2^X=0.747, R^2^Y=0.996, Q^2^=0.736 (PEDV-LW-J vs PEDV-LC-J);OPLS-DA (caecum ESI+): R^2^X=0.524, R^2^Y=0.995, Q^2^=0.549 (PEDV-LC-C vs Con-LC-C); R^2^X=0.571, R^2^Y=0.991, Q^2^=0.723 (PEDV-LW-C vs Con-LW-C); R^2^X=0.434, R^2^Y=0.977, Q^2^=0.541 (Con-LW-C vs Con-LC-C); R^2^X=0.524, R^2^Y=0.995, Q^2^=0.549 (PEDV-LW-C vs PEDV-LC-C);OPLS-DA (caecum ESI-): R^2^X=0.477, R^2^Y=0.99, Q^2^=0.653 (PEDV-LC-C vs Con-LC-C); R^2^X=0.75, R^2^Y=1, Q^2^=0.877 (PEDV-LW-C vs Con-LW-C); R^2^X=0.791, R^2^Y=0.997, Q^2^=0.651 (Con-LW-C vs Con-LC-C); R^2^X=0.608, R^2^Y=0.997, Q^2^=0.597 (PEDV-LW-C vs PEDV-LC-C).
