# Supplementary material for: Difference analysis of intestinal microbiota and metabolites in piglets of different breeds exposed to porcine epidemic diarrhea virus infection
Source: Front Microbiol. 2022 Nov 1;13:990642. doi: 10.3389/fmicb.2022.990642 (PMC9665409; doi:10.3389/fmicb.2022.990642)
Supplement: Supplementary file 1 [file Data_Sheet_1.ZIP › supplementary meterials/supplementary table 1.docx]

**SUPPLEMENTARY TABLE 1.** Information on metabolites differing between groups in the jejunum

| **Group** | **Metabolites** | **VIP value** | **FC value** | **P value** | **Type** |
| --- | --- | --- | --- | --- | --- |
| PEDV LC vs.Con LC group | 4-Aminobutyric acid | 1.29 | 5.49 | 0.007 | up |
|  | L-Citrulline | 2.86 | 3.84 | 0.029 | up |
|  | Hypoxanthine | 1.60 | 3.29 | 0.045 | up |
|  | 1,3,5(10)-Estratrien-3,17. beta. -diol 17-glucosiduronate | 2.08 | 6.66 | 0.006 | up |
|  | DL-2-Phosphoglycerate | 2.06 | 3.09 | 0.041 | up |
|  | Creatinine | 5.82 | 0.44 | 0.040 | down |
|  | NG, NG-dimethyl-L-arginine (ADMA) | 7.58 | 0.45 | 0.005 | down |
|  | Argininosuccinic acid | 2.12 | 0.34 | 0.011 | down |
|  | N-Acetyl-DL-methionine | 1.42 | 0.31 | 0.020 | down |
| PEDV LW vs. Con LW group | N1-Methyl-2-pyridone-5-carboxamide | 3.34 | 6.69 | <0.001 | up |
|  | 5-Methylcytosine | 1.30 | 2.91 | 0.046 | up |
|  | Allopurinol | 1.80 | 4.28 | <0.001 | up |
|  | N6-methyladenosine | 1.69 | 3.78 | 0.043 | up |
|  | L-Carnosine | 2.77 | 3.55 | 0.040 | up |
|  | (3-Carboxypropyl) trimethylammonium cation | 11.99 | 3.12 | 0.006 | up |
|  | 4-Hydroxybutanoic acid lactone | 1.57 | 2.52 | 0.016 | up |
|  | Urea | 1.67 | 2.18 | 0.040 | up |
|  | N-Oleoylethanolamine | 1.33 | 1.85 | <0.001 | up |
|  | Bisindolylmaleimide I | 1.85 | 2.98 | 0.009 | up |
|  | Indoxyl sulfate | 6.18 | 4.45 | 0.045 | up |
|  | 2-Oleoyl-1-stearoyl-sn-glycero-3-phosphoserine | 1.36 | 3.24 | 0.009 | up |
|  | L-Leucine | 2.31 | 0.50 | 0.033 | down |
|  | Cyclohexylamine | 5.03 | 0.39 | 0.045 | down |
|  | Dopamine | 1.27 | 0.39 | 0.022 | down |
|  | D-Proline | 3.61 | 0.68 | 0.008 | down |
|  | 1-Aminocyclopropanecarboxylic acid | 1.21 | 0.36 | 0.037 | down |
|  | S-Adenosylmethionine | 1.21 | 0.34 | 0.023 | down |
|  | 1-Palmitoyl-2-hydroxy-sn-glycero-3-phosphoethanolamine | 3.30 | 0.33 | 0.041 | down |
|  | N-Acetyl-D-Glucosamine 6-Phosphate | 1.10 | 0.19 | 0.030 | down |
|  | N-Acetyl-D-glucosamine | 2.82 | 0.17 | 0.002 | down |
|  | Met-Ala | 1.022 | 0.142 | 0.047 | down |
|  | Asp-Arg | 1.42 | 0.11 | 0.004 | down |
|  | Phe-Gly | 2.12 | 0.03 | 0.049 | down |
|  | L-Valine | 1.16 | 0.54 | 0.046 | down |
|  | L-Iditol | 1.03 | 0.29 | 0.025 | down |
|  | N-Acetyl-DL-methionine | 1.74 | 0.18 | 0.027 | down |
|  | Saccharin | 4.82 | 0.16 | 0.043 | down |
|  | Muramic acid | 1.91 | 0.16 | 0.001 | down |
|  | L-Gulonic gamma-lactone | 1.23 | 0.16 | 0.002 | down |
|  | D-Lyxose | 1.52 | 0.11 | 0.001 | down |
| PEDV LW vs. PEDV LC group | N1-Methyl-2-pyridone-5-carboxamide | 3.34 | 6.69 | 0.001 | up |
|  | Adenine | 3.17 | 6.21 | 0.004 | up |
|  | Pyridoxine | 1.29 | 4.68 | 0.027 | up |
|  | Dimethylaminopurine | 1.19 | 4,19 | 0.033 | up |
|  | 5-Methylcytosine | 1.29 | 2.91 | 0.046 | up |
|  | Oxyquinoline | 1.10 | 2.32 | <0.001 | up |
|  | Bisindolylmaleimide I | 1.85 | 2.98 | 0.009 | up |
|  | Taurine | 5.28 | 0.52 | 0.029 | down |
|  | Gly-Glu | 1.77 | 0.51 | 0.010 | down |
|  | gamma-L-Glutamyl-L-glutamic acid | 3.04 | 0.49 | 0.028 | down |
|  | 1-Methylhistidine | 1.85 | 0.40 | 0.043 | down |
|  | L-Pyroglutamic acid | 3.41 | 0.39 | 0.017 | down |
|  | L-Asparagine | 1.61 | 0.14 | 0.015 | down |
| Control LW vs. Con LC group | Pyridoxamine (PM) | 1.19 | 24.94 | 0.003 | up |
|  | Asp-Arg | 1.65 | 6.63 | 0.008 | up |
|  | D-Lyxose | 1.64 | 3.19 | 0.012 | up |
|  | N-Acetyl-DL-methionine | 2.03 | 3.04 | 0.048 | up |
|  | D-Proline | 3.61 | 0.68 | 0.007 | down |
|  | 1,2-dioleoyl-sn-glycero-3-phosphatidylcholine | 3.62 | 0.65 | 0.017 | down |
|  | NG, NG-dimethyl-L-arginine (ADMA) | 7.57 | 0.45 | 0.004 | down |
