# Supplementary material for: Difference analysis of intestinal microbiota and metabolites in piglets of different breeds exposed to porcine epidemic diarrhea virus infection
Source: Front Microbiol. 2022 Nov 1;13:990642. doi: 10.3389/fmicb.2022.990642 (PMC9665409; doi:10.3389/fmicb.2022.990642)
Supplement: Supplementary file 1 [file Data_Sheet_1.ZIP › supplementary meterials/supplementary table 2.docx]

**SUPPLEMENTARY TABLE 2.** Information on metabolites differing between groups in the cecum

| **Group** | **Metabolites** | **VIP value** | **FC value** | **P value** | **Type** |
| --- | --- | --- | --- | --- | --- |
| PEDV LC vs. Con LC group | Histamine | 2.57 | 5.28 | 0.047 | up |
|  | 1-Methylhistidine | 2.09 | 3.60 | 0.028 | up |
|  | 1-Myristoyl-sn-glycero-3-phosphocholine | 5.36 | 3.36 | 0.007 | up |
|  | 4-Aminobutyric acid | 1.10 | 3.09 | 0.035 | up |
|  | alpha-Linolenic acid | 1.92 | 1.63 | 0.030 | up |
|  | Hydroxyisocaproic acid | 12.92 | 5.05 | <0.001 | up |
|  | D-Proline | 5.37 | 0.37 | 0.033 | down |
| PEDV LW vs. Con LW group | N1-Methyl-2-pyridone-5-carboxamide | 3.31 | 5.68 | 0.035 | up |
|  | N2, N2-Dimethylguanosine | 1.13 | 5.27 | 0.044 | up |
|  | 1-Methylnicotinamide | 3.31 | 4.51 | 0.009 | up |
|  | Allopurinol | 2.44 | 3.86 | 0.031 | up |
|  | 4-Aminobutyric acid | 1.38 | 3.78 | <0.001 | up |
|  | 1-Methylhistidine | 3.31 | 4.51 | 0.009 | up |
|  | Betaine | 1.62 | 1.66 | 0.017 | up |
|  | L-Threonate | 1.07 | 1.73 | 0.012 | up |
|  | Dimethyl sulfone | 1.44 | 0.80 | 0.028 | down |
|  | 1-Stearoyl-2-hydroxy-sn-glycero-3-phosphocholine | 11.07 | 0.56 | 0.041 | down |
|  | Adenine | 3.64 | 0.37 | 0.032 | down |
|  | Acetylcholine | 1.73 | 0.06 | 0.043 | down |
|  | Beta-D-Fructose 2-phosphate | 1.18 | 0.06 | 0.045 | down |
| PEDV LW vs. PEDV LC group | N1-Methyl-2-pyridone-5-carboxamide | 3.43 | 5.40 | 0.036 | up |
|  | 1-Methylnicotinamide | 3.33 | 2.79 | 0.019 | up |
|  | Creatinine | 13.10 | 2.59 | 0.046 | up |
|  | L-Sorbose | 1.08 | 2.01 | 0.047 | up |
|  | 1-Myristoyl-sn-glycero-3-phosphocholine | 5.08 | 0.29 | 0.005 | down |
|  | 1-Oleoyl-L-. alpha. -lysophosphatidic acid | 2.36 | 0.25 | 0.040 | down |
|  | 1-Palmitoylglycerol | 1.44 | 0.21 | 0.014 | down |
|  | L-Gulonic gamma-lactone | 1.09 | 0.45 | 0.016 | down |
|  | Capric acid | 1.11 | 0.44 | 0.028 | down |
|  | Hydroxyisocaproic acid | 12.84 | 0.34 | 0.022 | down |
| Control LW vs. Con LC group | L-Leucine | 4.38 | 0.48 | 0.040 | down |
