# Supplementary figures and images for: Difference analysis of intestinal microbiota and metabolites in piglets of different breeds exposed to porcine epidemic diarrhea virus infection
Source: Front Microbiol. 2022 Nov 1;13:990642. doi: 10.3389/fmicb.2022.990642 (PMC9665409; doi:10.3389/fmicb.2022.990642)

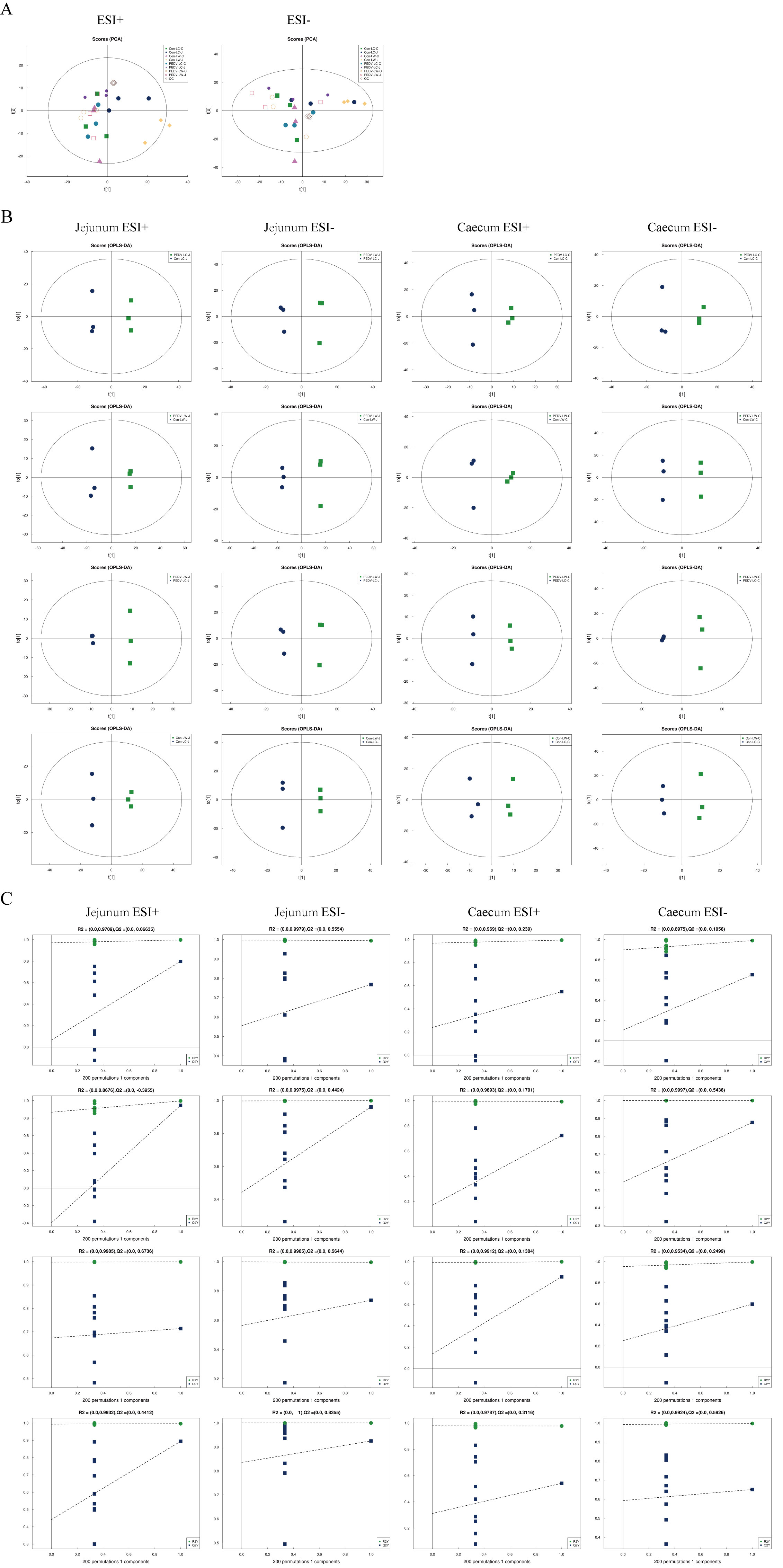

Supplement: Supplementary file 1 [file Data_Sheet_1.ZIP › supplementary meterials/supplementary figure 1.jpg]
